# Biomechanical and Tomographic Outcomes in Pediatric Keratoconus Treated with Conventional Epithelium-Off Corneal Collagen Cross-Linking

**DOI:** 10.3390/medicina62061145

**Published:** 2026-06-12

**Authors:** Radu-Nicolae Pop, Patricia Ariadna Nicula, Cristina Ariadna Nicula, Dorin Vasile Nicula, Bianca Pop

**Affiliations:** 1 OCULENS Ophthalmology Clinic, 400501 Cluj-Napoca, Romania; radupop1985@yahoo.com (R.-N.P.); cristinanicula65@yahoo.com (C.A.N.); niculadorin60@yahoo.com (D.V.N.); 2 Faculty of Medicine, “Lucian Blaga” University, 550169 Sibiu, Romania; 3 Department of Oral and Maxillofacial Surgery and Radiology, Faculty of Dentistry, “Iuliu Hațieganu” University of Medicine and Pharmacy, 400012 Cluj-Napoca, Romania; 4 Surgical Department, University of Agricultural Sciences and Veterinary Medicine, 400372 Cluj-Napoca, Romania

**Keywords:** pediatric keratoconus, corneal collagen cross-linking, Pentacam, Corvis ST, corneal tomography, corneal biomechanics, adolescents

## Abstract

*Background and Objectives*: Pediatric keratoconus may progress rapidly, and treatment decisions are often made before prolonged observation is possible. This study evaluated 24-month visual, tomographic, pachymetric, and biomechanical outcomes after conventional epithelium-off corneal collagen cross-linking (CXL) using Pentacam tomography and Corvis ST dynamic Scheimpflug analysis. *Materials and Methods*: This single-center observational longitudinal cohort included 28 eyes of 23 patients aged 13–18 years treated at the OCULENS Clinic, Cluj-Napoca, Romania, between 2019 and 2023. Because the study had no untreated or alternative-treatment control group, postoperative changes were interpreted as associations after CXL rather than as proof of causality. Baseline, 6-, 12-, and 24-month values were analyzed for UCVA, BCVA, Kmax, thinnest pachymetry, SP-A1, deformation amplitude (DA), first and second applanation times (A1T and A2T), highest concavity (HC) radius, and BAD-D. Repeated-measures ANOVA was used after assessment of within-eye difference normality; findings were interpreted cautiously because the analysis was eye-based, the cohort was small, and multiple outcomes were examined. *Results*: UCVA improved from 0.53 ± 0.16 to 0.44 ± 0.16 logMAR and BCVA from 0.31 ± 0.11 to 0.25 ± 0.11 logMAR (both *p* < 0.001). Mean Kmax decreased modestly from 54.36 ± 3.11 D to 53.41 ± 2.79 D, while SP-A1 increased from 84.69 ± 4.75 to 97.39 ± 5.11 (both *p* < 0.001). Thinnest pachymetry showed early postoperative thinning followed by partial recovery by 24 months. DA decreased, A1T increased, A2T decreased, HC radius increased, and BAD-D decreased significantly. Kmax and SP-A1 were inversely correlated at all visits (r = −0.714 to −0.773; all *p* < 0.001), but these correlations were considered exploratory. Post-24-month retreatment and keratoplasty-related events were recorded descriptively and were not included in the formal 24-month model. *Conclusions*: Within the prespecified 24-month analytic window, conventional epithelium-off CXL was associated with stabilization, modest visual and tomographic improvement, and a concordant biomechanical stiffening signal. The results should be interpreted as cautious observational findings rather than definitive evidence of long-term stability, because of the small sample, eye-level analysis, absence of a control group, limited follow-up, and lack of formal repeatability testing.

## 1. Introduction

Keratoconus is a bilateral, usually asymmetric ectatic corneal disorder characterized by progressive stromal thinning, increasing irregular astigmatism, and loss of optical quality that may culminate in corneal scarring or transplantation in advanced disease [1]. Contemporary ectasia frameworks emphasize that diagnosis and follow-up should not rely on anterior curvature alone, but instead should integrate tomographic and biomechanical information within the broader clinical context [2]. This approach is biologically coherent because the shape and load-bearing behavior of the cornea arise from a highly ordered stromal collagen architecture whose disruption alters both transparency and mechanical stability [3]. Eye rubbing, ocular allergy, and chronic surface inflammation are particularly relevant in this setting because they are repeatedly associated with keratoconus development and progression [4].

The pediatric phenotype is clinically distinct from adult disease. Children and adolescents often present after a period of silent progression and may show aggressive steepening, faster thinning, and earlier functional deterioration than older patients [5,6]. The biological rationale for timely intervention is that a young ectatic cornea may have limited biomechanical reserve: continued stromal thinning and collagen-disorganization can amplify irregular astigmatism, reduce contact-lens tolerance, and narrow the safety margin for later procedures. Therefore, pediatric management often prioritizes the prevention of further structural loss rather than waiting for large numerical changes to accumulate.

The rationale for early CXL is therefore preventive and biomechanical rather than refractive. CXL is expected to increase stromal resistance before further ectatic deformation reduces optical quality or leaves insufficient tissue for standard treatment. At the same time, early treatment decisions in children are difficult because diagnostic progression can be masked or exaggerated by cooperation, tear-film instability, allergic flares, eye-rubbing behavior, and changes in correction. This clinical tension explains why pediatric studies should report both mean outcomes and methodological uncertainty.

Corneal collagen cross-linking was developed to increase stromal biomechanical resistance through a riboflavin-mediated photochemical reaction induced by ultraviolet A irradiation [7,8]. Conventional epithelium-off CXL remains the reference protocol because its laboratory basis, long-term adult clinical evidence, and reproducibility are more mature than those of alternative approaches [9,10]. In pediatric keratoconus, the literature broadly supports the effectiveness of standard epithelium-off treatment while also showing that outcomes are not uniform and that some eyes continue to progress despite apparently adequate treatment [11,12,13,14,15,16,17,18,19,20,21,22,23,24,25]. This point is especially important when interpreting short- and mid-term pediatric results, because apparent stabilization at 1 or 2 years does not always exclude later recurrence of progression.

Although transepithelial and other modified protocols have been studied, epithelium-off treatment generally remains the most dependable benchmark for meaningful structural stabilization [26,27]. Pentacam tomography allows for the longitudinal assessment of corneal curvature, posterior elevation, and pachymetric behavior, whereas Corvis ST adds a dynamic biomechanical signal that may help determine whether the postoperative cornea has become less deformable. However, both modalities have limitations in children and adolescents. Tomographic and biomechanical values can be affected by fixation, blinking, tear-film instability, corneal hydration, epithelial remodeling, intraocular pressure, and acquisition quality. Consequently, isolated small changes in Kmax, pachymetry, BAD-D, DA, or SP-A1 should not be interpreted as definitive progression or definitive therapeutic success without clinical correlation [28,29,30,31,32,33,34,35].

The aim of the present study was to describe 24-month outcomes after conventional epithelium-off CXL in 28 treated eyes from 23 pediatric patients using Pentacam tomography together with Corvis ST-derived biomechanical parameters, including SP-A1, DA, A1T, A2T, HC radius, and BAD-D. In response to the limitations inherent to a small single-center cohort, the study was deliberately framed as observational and hypothesis-generating, with emphasis on effect direction, clinical magnitude, measurement variability, and the need for longer patient-level follow-up rather than on definitive causal inference.

## 2. Materials and Methods

### 2.1. Study Design, Setting, and Analytic Window

This was a single-center observational longitudinal cohort study of 28 eyes from 23 pediatric patients aged 13–18 years who underwent conventional epithelium-off CXL for progressive keratoconus. The patients were treated at the Cluj-Napoca OCULENS Clinic between 2019 and 2023. During this interval, 167 eyes underwent treatment for keratoconus at the center, of which 28 eyes were pediatric keratoconus cases, corresponding to 16.8% of all treated keratoconus eyes.

The study did not include an untreated control group or an alternative-treatment comparator. Consequently, the statistical results describe changes observed after CXL in this clinical cohort and should not be interpreted as definitive proof that all observed changes were caused exclusively by CXL. Natural variability, changes in eye rubbing or allergy control, contact lens behavior, regression to the mean, and other unmeasured clinical factors cannot be fully excluded.

Prespecified study visits were baseline, 6 months, 12 months, and 24 months. Eyes that later required retreatment or keratoplasty evaluation were retained in the 24-month analysis if they completed the scheduled 24-month visit before the late event. Their post-24-month clinical course was recorded separately as descriptive safety and durability information and was not included in the formal repeated-measures model. Therefore, these late events did not change eligibility and were not used to support the 24-month statistical conclusions.

### 2.2. Eligibility Criteria

Inclusion criteria. Eyes were eligible when all of the following conditions were met.

- Disease and progression criteria. A clinical diagnosis of keratoconus stages I to IV according to the Amsler–Krumeich classification was required, based on refracto-keratometric findings, corneal tomography, and slit-lamp examination. Diagnostic features included at least one of the following: asymmetric inferior–superior steepening, skewed radial axis, posterior elevation abnormalities, or stromal thinning with or without a Fleischer ring or Vogt striae. Because pediatric keratoconus is known to progress more rapidly than adult disease, treatment was recommended without delaying intervention for prolonged observation once the diagnosis and high likelihood of progression were established. When documented progression was available, it was defined within 12 months by at least one of the following: an increase in Kmax of ≥1.0 D, an increase in manifest cylinder of ≥1.0 D, a reduction in minimal pachymetry of ≥10 µm, or deterioration of BCVA by ≥0.1 logMAR not attributable to another ocular pathology. Eyes with Kmax ≥ 65.0 D were not included in order to avoid extremely advanced ectasia with poor visual potential and increased procedural uncertainty; this safety-based criterion also narrows the applicability of the results to very advanced pediatric keratoconus.
- Anatomical and safety criteria. Eligible eyes were required to have a minimal stromal thickness of at least 400 µm after epithelial removal and riboflavin saturation, a clear central cornea without visually significant scarring, and endothelial cell density within the age-appropriate normal range on specular microscopy. Eyes with active ocular surface disease, including severe dry eye, sicca keratoconjunctivitis, or Sjögren syndrome, were not considered suitable for treatment.
- General criteria. Additional inclusion criteria were age ≤ 18 years, ability to comply with scheduled follow-up visits through 24 months, and no previous CXL in the study eye.

Exclusion criteria. Eyes were excluded if any of the following conditions were present: prior corneal surgery, including keratoplasty, refractive surgery, or intracorneal ring implantation; active ocular infection or inflammation; central corneal scarring or opacification affecting the visual axis; hydrops or a history of acute corneal hydrops; severe dry eye or ocular surface disease impairing epithelial healing; previous herpetic keratitis; autoimmune or connective tissue disorders affecting corneal healing; endothelial dysfunction; intraocular pressure > 21 mmHg or glaucoma with optic nerve damage; pregnancy or breastfeeding at the time of the procedure; systemic collagen vascular disease; immunosuppressive therapy; diabetes associated with impaired wound healing; postoperative medication non-adherence; or inability to ensure the safe completion of UVA exposure. These criteria intentionally selected eyes in which standard epithelium-off CXL could be performed safely, but they also limit generalizability to very advanced, scarred, or very thin pediatric keratoconus.

### 2.3. Clinical, Tomographic, and Biomechanical Measurements

Ocular examination before treatment included uncorrected visual acuity (UCVA) and best-corrected visual acuity (BCVA), both recorded on the logMAR scale; refractometry and keratometry; slit-lamp biomicroscopy; intraocular pressure measurement (Applano-T, (CSO (Costruzione Strumenti Oftalmici) R Type Applanation Tonometer (A900), Florence, Italy) in cooperative patients, iCare IC 100 tonometer, (Icare Finland Oy, Vantaa, Finland) in less cooperative patients, and biomechanically corrected IOP when available); endothelial cell count by specular microscopy; Pentacam corneal tomography including pachymetry; and Corvis ST biomechanical assessment.

Pentacam and Corvis ST measurements were obtained by trained clinic personnel according to manufacturer recommendations and routine pediatric cooperation standards. Fixation quality, centration, and device quality specifications were reviewed at acquisition. Scans with poor fixation, blinking, marked decentration, or unacceptable quality flags were repeated. When more than one clinically acceptable acquisition was available, the best-quality acquisition retained in the medical record for clinical decision-making was used for the study database; values were not averaged across multiple scans. A formal within-session repeatability, inter-operator repeatability, or test–retest substudy was not performed. This may introduce measurement bias and reduces confidence in small changes near the expected repeatability limits of tomography and dynamic Scheimpflug imaging, particularly in pediatric patients.

Corvis ST dynamic Scheimpflug measurements included deformation amplitude (DA, mm), representing maximal apical displacement; first applanation time (A1T, ms) and second applanation time (A2T, ms); stiffness parameter at first applanation (SP-A1), used as an estimate of corneal stiffness; and highest concavity (HC) radius (mm), which reflects the curvature radius at the corneal apex during the highest concavity phase. Pentacam-derived BAD-D was reviewed as a composite tomographic ectasia index. Measurements were collected at the baseline and at 6, 12, and 24 months.

### 2.4. Cross-Linking Procedure

The cross-linking technique was performed under sterile operating-room conditions using the conventional Dresden epithelium-off protocol. After the riboflavin 0.1–dextran 20% solution was prepared and the UVA illuminator output was verified according to the device calibration procedure, topical anesthesia with proparacaine (Alcaine) was administered as 3–4 drops over the 15–20 min preceding treatment. Manual corneal de-epithelialization was performed over an approximately 9 mm central zone using a sterile epithelial spatula under the operating microscope.

Riboflavin 0.1% was instilled every 3 min for 30 min to achieve stromal saturation. Before UVA exposure, stromal riboflavin saturation was confirmed clinically by homogeneous yellow stromal staining and riboflavin presence in the anterior chamber. Corneal thickness was checked to confirm that the safety threshold of at least 400 µm was maintained after epithelial removal and riboflavin saturation. Cases below this threshold were not treated under the standard protocol.

The cornea was then exposed to UVA irradiation at 3 mW/cm^2^ for 30 min, corresponding to a total fluence of 5.4 J/cm^2^. The UVA beam was centered on the corneal apex, the working distance and alignment were maintained throughout treatment, and riboflavin instillation continued every 3 min during irradiation. Device output was verified according to the clinical calibration procedure before treatment; however, independent external irradiance mapping or spatial beam-profile verification was not performed for this retrospective analysis. This limits reproducibility assessment and prevents retrospective confirmation that the delivered irradiance distribution was identical across all eyes.

### 2.5. Outcomes and Statistical Analysis

The primary outcomes were UCVA, BCVA, Kmax, thinnest pachymetry, and SP-A1. Secondary outcomes were DA, A1T, A2T, HC radius, and BAD-D. The primary clinical interpretation focused on stabilization and the direction of change rather than on refractive reversal, because pediatric CXL is primarily intended to halt or slow ectatic progression.

Continuous variables were summarized as mean ± standard deviation. Longitudinal change across the baseline, 6 months, 12 months, and 24 months was assessed using repeated-measures analysis of variance. To justify parametric repeated-measures testing, the distribution of within-eye differences was assessed by Shapiro–Wilk testing and visual inspection of Q–Q plots for the main outcomes. When no major deviation from normality was identified, repeated-measures ANOVA was retained. Sphericity was evaluated, and Greenhouse–Geisser correction was planned if the sphericity assumptions were not met. Pearson correlation was used to assess the relationship between Kmax and SP-A1 at each time point as an exploratory structure–function analysis.

Because numerous variables were assessed over time, statistical significance was interpreted with emphasis on the prespecified primary outcomes and on the concordance of the overall clinical, tomographic, and biomechanical pattern. Holm correction was considered for the family of longitudinal outcome tests; as all repeated-measures *p* values were <0.001, the principal longitudinal findings remained statistically significant after conservative adjustment. However, *p* values should still be interpreted cautiously because the analysis was eye-based rather than patient-based, the cohort was small, multiple outcomes were examined, and statistical significance does not automatically indicate a large clinical effect.

The analysis was performed at the eye level. Since 23 patients contributed 28 eyes, bilateral-eye dependence could not be fully adjusted by mixed-effects or generalized estimating-equation models without overfitting this small dataset. Therefore, the *p* values may overestimate statistical precision and should be viewed as descriptive inferential signals rather than as confirmatory patient-level evidence. This limitation is emphasized in Section 4, and future larger studies should use patient-clustered statistical models.

Figures were recreated at high resolution from anonymized aggregate study data. Error bars represent standard deviations to display between-eye variability, and not standard errors. A descriptive change-magnitude figure was added to make the baseline-to-24-month effect sizes transparent. These figures are visual summaries only and are not intended to imply individual-eye trajectories or causal effects.

## 3. Results

The analytic cohort comprised 28 treated eyes from 23 pediatric patients. Mean age at treatment was 16.0 ± 1.6 years (range 13–18), atopy was recorded in 18 eye-level records (64.3%), and stage 3 or 4 keratoconus accounted for 18 eyes (64.3%), indicating that the cohort was weighted toward moderate-to-advanced ectasia. Baseline eye-level characteristics are summarized in Table 1. This stage distribution is important when interpreting generalizability, because early-stage responses were underrepresented and eyes with Kmax ≥ 65.0 D were not included.

Assumption checking did not identify major departures from normality in the main within-eye differences, supporting the use of parametric repeated-measures testing for the planned analysis. All repeated-measures longitudinal *p* values were <0.001 after the consideration of multiple longitudinal endpoints; nevertheless, the small cohort, bilateral-eye structure, and aggregate nature of the data mean that the clinical relevance of each statistically significant result must be considered separately.

**Figure 1:**
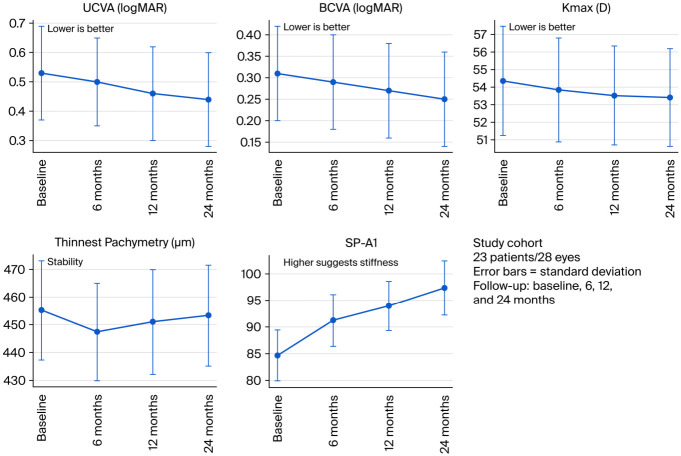
Primary visual, tomographic, pachymetric, and biomechanical outcomes from the baseline to 24 months. Error bars represent standard deviations. Lower logMAR and Kmax values indicate improvement or flattening, whereas higher SP-A1 values are compatible with greater resistance to deformation. The visual and Kmax changes are modest in absolute magnitude and should be interpreted as stabilization-associated aggregate changes rather than major refractive reshaping.

Visual acuity improved progressively over follow-up. Mean UCVA improved from 0.53 ± 0.16 logMAR at the baseline to 0.50 ± 0.15 at 6 months, 0.46 ± 0.16 at 12 months, and 0.44 ± 0.16 at 24 months (RM-ANOVA F = 98.02, *p* < 0.001, partial η^2^ = 0.784). Mean BCVA improved from 0.31 ± 0.11 to 0.25 ± 0.11 logMAR over the same interval (F = 125.66, *p* < 0.001, partial η^2^ = 0.823). The absolute 24-month visual gains were modest (−0.08 logMAR for UCVA and −0.06 logMAR for BCVA), and therefore best interpreted as functional improvement accompanying stabilization rather than as a large refractive rehabilitation effect (Figure 1 and Table 2).

Pentacam tomography showed a modest flattening pattern. Mean Kmax decreased from 54.36 ± 3.11 D at the baseline to 53.84 ± 2.96 D at 6 months, 53.52 ± 2.82 D at 12 months, and 53.41 ± 2.79 D at 24 months (F = 127.65, *p* < 0.001, partial η^2^ = 0.825). Thinnest pachymetry decreased from 455.3 ± 17.9 µm to 447.5 ± 17.6 µm at 6 months, followed by partial return toward the baseline at 451.1 ± 18.9 µm at 12 months and 453.4 ± 18.2 µm at 24 months (F = 71.43, *p* < 0.001, partial η^2^ = 0.726). The mean 24-month changes relative to baseline were −0.95 D for Kmax and −1.9 µm for thinnest pachymetry. This pattern is compatible with tomographic stabilization and limited flattening, but it should not be interpreted as substantial anatomic reversal (Figure 1 and Table 2).

**Figure 2:**
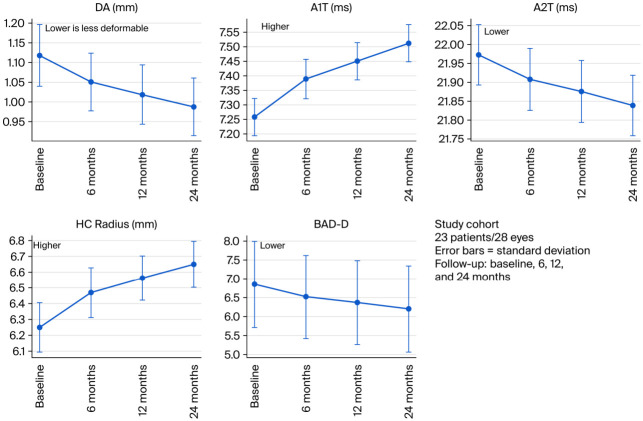
Secondary Corvis ST and BAD-D outcomes from baseline to 24 months. The direction of change across DA, A1T, A2T, HC radius, and BAD-D was concordant with reduced corneal deformability and tomographic stabilization after CXL; however, the figure is an aggregate summary and does not replace individual clinical follow-up.

Corvis ST demonstrated a concordant postoperative signal compatible with reduced corneal deformability. Mean SP-A1 increased from 84.69 ± 4.75 at the baseline to 91.25 ± 4.89 at 6 months, 93.96 ± 4.62 at 12 months, and 97.39 ± 5.11 at 24 months (F = 537.51, *p* < 0.001, partial η^2^ = 0.952). In parallel, DA decreased from 1.118 ± 0.078 to 0.988 ± 0.073 mm (F = 1330.64, *p* < 0.001, partial η^2^ = 0.980), A1T increased from 7.258 ± 0.064 to 7.512 ± 0.064 ms (F = 1286.00, *p* < 0.001, partial η^2^ = 0.979), A2T decreased from 21.973 ± 0.080 to 21.839 ± 0.080 ms (F = 305.10, *p* < 0.001, partial η^2^ = 0.919), HC radius increased from 6.251 ± 0.156 to 6.650 ± 0.146 mm (F = 879.77, *p* < 0.001, partial η^2^ = 0.970), and BAD-D decreased from 6.854 ± 1.141 to 6.203 ± 1.139 (F = 507.22, *p* < 0.001, partial η^2^ = 0.949). These aggregate changes are summarized in Figure 2 and Table 3.

**Figure 3:**
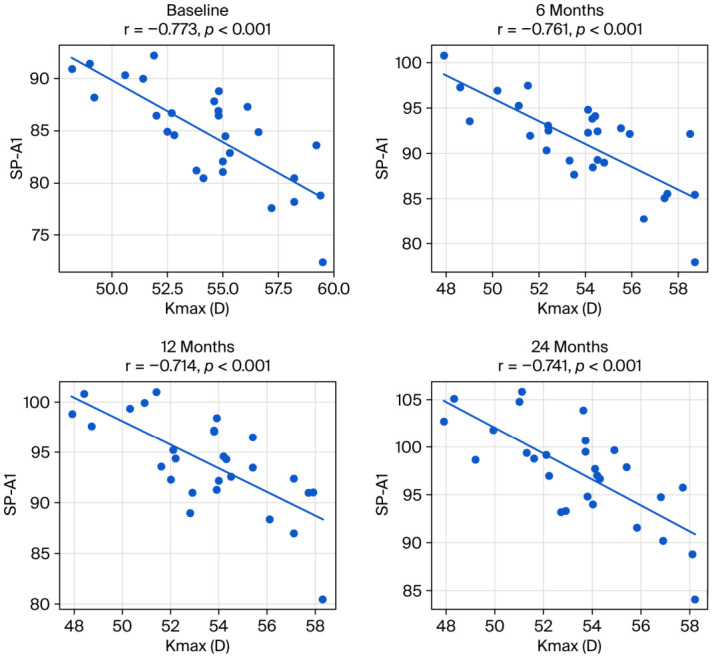
Exploratory scatterplots showing the inverse association between Kmax and SP-A1 at each visit. Across all time points, steeper corneas tended to demonstrate lower stiffness parameter values. These plots support internal consistency between tomographic severity and biomechanical behavior, but the correlations are preliminary and should not be interpreted as proof of causality or individual predictive value.

**Figure 4:**
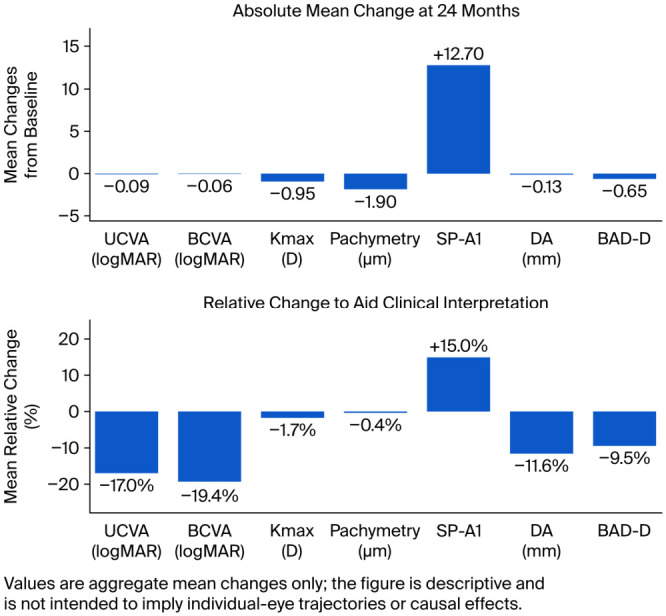
Descriptive baseline-to-24-month change magnitude for selected outcomes. The bars summarize aggregate mean changes only. These were added to make the modest absolute magnitude of several statistically significant outcomes transparent and should not be interpreted as individual-eye trajectories or causal evidence. Corvis ST SP-A1 increases after corneal collagen cross-linking (CXL), because CXL is intended to increase corneal resistance to deformation. SP-A1 means stiffness parameter at first applanation. A higher SP-A1 indicates that, during the Corvis ST air-puff deformation, the cornea shows greater resistance to inward displacement.

The biomechanical and tomographic signals were associated but should be considered exploratory. Kmax and SP-A1 showed inverse correlations at baseline (r = −0.773, *p* < 0.001), 6 months (r = −0.761, *p* < 0.001), 12 months (r = −0.714, *p* < 0.001), and 24 months (r = −0.741, *p* < 0.001), indicating that steeper corneas tended to have lower stiffness parameter values. These correlations support biological plausibility but do not prove causation or predictive value. The repeated-measures ANOVA summary and longitudinal correlations are presented in Figure 3 and Table 4. To make the modest absolute magnitude of several changes more visible, baseline-to-24-month mean changes are summarized descriptively in Figure 4.

Regarding adverse clinical evolution beyond the predefined 24-month analytic window, subsequent review of clinical follow-up records showed that three eyes required retreatment at approximately 3 years because of renewed progression, and one patient later developed an indication for keratoplasty. These eyes were not excluded from the 24-month analysis if their 24-month data were complete; however, post-24-month values and post-retreatment data were not entered into the repeated-measures model. These events are therefore reported only as descriptive late clinical observations and are not used to support the formal 24-month conclusions.

## 4. Discussion

The present study showed that conventional epithelium-off CXL in pediatric keratoconus was associated with visual improvement, modest tomographic flattening, and a concordant Corvis ST stiffening pattern over 24 months. The most clinically important interpretation is not that the cornea was markedly reshaped, but that the treated eyes showed no mean pattern of progressive steepening during the scheduled follow-up. This distinction is essential in pediatric keratoconus, where the principal goal of treatment is stabilization and the prevention of further visual loss rather than refractive normalization.

The direction of our findings is broadly consistent with previous pediatric CXL studies and reviews, which generally report stabilization or modest improvement after standard epithelium-off treatment while also acknowledging that pediatric eyes may continue to progress after treatment [11,12,13,14,15,16,17,18,19,20,21,22,23,24,25]. Earlier pediatric series, including studies by Chatzis and Hafezi, Vinciguerra and colleagues, and Zotta and colleagues, helped establish the feasibility and short-term effectiveness of CXL in children and adolescents [11,12,13]. Longer follow-up reports and systematic reviews later emphasized that the benefit may persist in many eyes but is not absolute, especially in younger patients or in more aggressive forms of disease [14,15,18,20,21,24,25]. Our 24-month results fit within this pattern: the average treated eye stabilized or improved, but the study design does not allow for an estimation of how the same eyes would have evolved without treatment.

The magnitude of the tomographic effect deserves careful interpretation. The mean Kmax reduction of approximately 1 D is statistically significant and clinically favorable but modest. In a refractive procedure, such a change would be considered limited; in pediatric keratoconus, however, absence of further mean steepening may still be clinically meaningful because untreated progressive disease may otherwise lead to increasing irregular astigmatism, contact lens intolerance, scarring, or eventual keratoplasty. The same principle applies to the visual results. The UCVA and BCVA improvements of approximately 0.08 and 0.06 logMAR, respectively, are small and may be influenced by measurement variability, ocular surface stability, optical correction, and pediatric cooperation. Therefore, these visual gains should be interpreted as supportive functional signals, not as definitive proof of treatment efficacy.

The clearest postoperative signal in this cohort was biomechanical. SP-A1 increased progressively, DA decreased, A1T increased, A2T decreased, HC radius increased, and BAD-D decreased. This coordinated pattern is more persuasive than any single parameter because it points in the same direction across different deformation descriptors. It is compatible with the biological rationale of CXL as a strengthening procedure that reduces corneal deformability under dynamic loading. Nevertheless, the absence of independent irradiance mapping and formal repeatability testing means that the magnitude of the biomechanical change should be interpreted with appropriate caution. Previous Corvis ST studies have similarly suggested that biomechanical parameters may provide useful information after CXL and may complement tomography in monitoring ectatic disease [28,29,30,31,32,33,34,35].

The inverse correlation between Kmax and SP-A1 at every time point also supports internal consistency: steeper corneas tended to have lower stiffness parameter values. Nevertheless, this relationship should not be overinterpreted. Correlation does not imply causation, and the analysis cannot determine whether higher stiffness directly caused lower Kmax, whether both reflect underlying disease severity, or whether additional factors such as corneal thickness, intraocular pressure, hydration, and measurement noise contributed. The clinical implication is that combined tomographic-biomechanical follow-up may be more informative than either modality alone, but larger prospective datasets are needed before using these correlations for individual risk prediction.

The pachymetric pattern also requires clinical nuance. The early postoperative thinning at 6 months followed by partial return toward the baseline at 12 and 24 months may reflect stromal compaction, epithelial remodeling, hydration changes, measurement repeatability, or a combination of these mechanisms. Therefore, the pachymetric course should be described as a pattern compatible with postoperative remodeling and stabilization at the aggregate level, not as definitive proof of structural recovery. The near-baseline 24-month thinnest pachymetry value, together with Kmax flattening and the biomechanical signal, is reassuring in the mean cohort; however, pachymetry remains vulnerable to measurement variability, hydration status, epithelial remodeling, and fixation quality, which are particularly relevant in pediatric patients.

Age and stage may influence pediatric CXL outcomes, but this study was not powered to provide reliable age- or stage-stratified conclusions. The age range was relatively narrow, with a mean age of 16 years, and the cohort was weighted toward stage 3 and 4 keratoconus. These features limit extrapolation to younger children, to very early disease, and to very advanced eyes excluded for safety reasons. In clinical practice, younger age, atopy, eye rubbing, and more advanced presentation are often considered risk markers for progression, and future studies should formally model these variables. In the present dataset, subgroup analysis by age or keratoconus stage would have been underpowered and potentially misleading; therefore, the manuscript reports the stage distribution transparently rather than presenting unstable subgroup claims.

The late events are important to report transparently even though they occurred beyond the formal 24-month endpoint. Three eyes required retreatment at approximately 3 years, and one patient later developed an indication for keratoplasty. These cases were not used to modify the 24-month statistical model and are not presented as a formal long-term outcome analysis. Their value is descriptive: they indicate that a favorable 24-month course cannot be assumed to represent durable long-term stability in every pediatric eye. This observation reinforces the need for continued clinical surveillance but should not be interpreted as an analyzed 3-year outcome of the present study.

Several limitations should be stated explicitly. First, the cohort was small, with only 28 eyes from 23 patients, and included bilateral treatment in some patients. Because the analysis was performed at eye level, the results may overestimate statistical precision. Second, the single-center observational design and absence of a control group prevent definitive attribution of all changes to CXL alone. Third, selection criteria required sufficient thickness and excluded very advanced eyes with Kmax ≥ 65 D, central scarring, or inadequate safety margins, limiting generalizability to more severe pediatric keratoconus; the small number of stage 1–2 eyes also limits inference about early-stage disease. Fourth, numerous parameters were analyzed over multiple time points. Although the principal findings remained significant after conservative consideration of multiple testing and the direction of change was biologically coherent, false-positive risk cannot be fully eliminated. Fifth, tomography and biomechanics were obtained under routine clinical conditions; unacceptable scans were repeated, but values were not averaged across multiple acquisitions, and a formal within-session or inter-operator repeatability analysis was not available. Sixth, device calibration was verified clinically, but independent irradiance mapping and spatial beam-profile documentation were not available retrospectively, which limits procedural reproducibility assessment.

The main future direction is a larger, multicenter, patient-level study with longer standardized follow-up. Such a study should use mixed-effects or generalized estimating-equation models to account for bilateral-eye clustering, prespecify a limited number of primary outcomes, incorporate correction for multiple comparisons, and include age-, stage-, atopy-, and eye-rubbing-based subgroup analysis. A 5- to 10-year follow-up design would be particularly valuable in pediatric keratoconus because late progression may become evident only after the period usually covered by short clinical studies. Standardized repeated tomography and Corvis ST acquisition, ideally with averaged measurements or repeatability thresholds, would also improve confidence in whether small changes are clinically meaningful or reflect measurement variability. The present study cannot answer these subgroup and long-term durability questions definitively, but it identifies them as clinically important priorities.

## 5. Conclusions

Within the prespecified 24-month analytic window, conventional epithelium-off CXL in this selected 28-eye pediatric keratoconus cohort was associated with stabilization of progressive disease, modest improvement in UCVA and BCVA, modest Kmax flattening, near-baseline pachymetry after early postoperative thinning, and a concordant Corvis ST pattern characterized by increased SP-A1 and reduced deformation amplitude. These conclusions are restricted to outcomes formally analyzed through 24 months. Because the study was small, single-center, uncontrolled, and eye-based without clustering adjustment, the findings should be interpreted as cautious observational associations rather than definitive evidence of treatment efficacy. The results apply primarily to selected pediatric eyes with adequate corneal thickness and without extremely advanced ectasia, and require confirmation in larger prospective patient-level studies with standardized acquisition and repeatability protocols.

Tables present the baseline characteristics, longitudinal outcomes, statistical summaries, and explanatory notes intended to distinguish statistical significance from clinical magnitude.

All figures were generated from aggregate, anonymized study data. No patient-identifying information is displayed. Error bars represent standard deviations rather than standard errors in order to visualize between-eye dispersion. The additional change-magnitude figure was included to clarify that several statistically significant outcomes had modest absolute clinical changes.

## Figures and Tables

**Table 1:** Cohort profile and baseline eye-level characteristics.

Variable	Value
Study cohort	23 patients/28 treated eyes
Age at treatment, years	16.0 ± 1.6 (range 13–18)
Atopy	18/28 eyes (64.3%)
Keratoconus stage 1	3 eyes (10.7%)
Keratoconus stage 2	7 eyes (25.0%)
Keratoconus stage 3	11 eyes (39.3%)
Keratoconus stage 4	7 eyes (25.0%)
Baseline UCVA (logMAR)	0.53 ± 0.16
Baseline BCVA (logMAR)	0.31 ± 0.11
Baseline Kmax (D)	54.36 ± 3.11
Baseline SP-A1	84.69 ± 4.75
Baseline thinnest pachymetry (µm)	455.3 ± 17.9

Note: The cohort was weighted toward moderate-to-advanced ectasia, with stage 3 or 4 disease in 64.3% of treated eyes.

**Table 2:** Primary longitudinal outcomes from the baseline to 24 months (mean ± SD).

Parameter	Baseline	6 Months	12 Months	24 Months	Delta, 24 Months-Baseline
UCVA (logMAR)	0.53 ± 0.16	0.50 ± 0.15	0.46 ± 0.16	0.44 ± 0.16	−0.08
BCVA (logMAR)	0.31 ± 0.11	0.29 ± 0.11	0.27 ± 0.11	0.25 ± 0.11	−0.06
Kmax (D)	54.36 ± 3.11	53.84 ± 2.96	53.52 ± 2.82	53.41 ± 2.79	−0.95
SP-A1	84.69 ± 4.75	91.25 ± 4.89	93.96 ± 4.62	97.39 ± 5.11	+12.70
Thinnest pachymetry (µm)	455.3 ± 17.9	447.5 ± 17.6	451.1 ± 18.9	453.4 ± 18.2	−1.9

Note: Visual and Kmax changes were statistically significant but modest in absolute magnitude; these are interpreted as supportive of stabilization rather than as large refractive or anatomic remodeling.

**Table 3:** Secondary Corvis ST and Pentacam indices across follow-up (mean ± SD).

Parameter	Baseline	6 Months	12 Months	24 Months	Delta, 24 Months-Baseline
DA (mm)	1.118 ± 0.078	1.051 ± 0.073	1.019 ± 0.075	0.988 ± 0.073	−0.130
A1T (ms)	7.258 ± 0.064	7.389 ± 0.068	7.450 ± 0.064	7.512 ± 0.064	+0.254
A2T (ms)	21.973 ± 0.080	21.908 ± 0.082	21.876 ± 0.082	21.839 ± 0.080	−0.133
HC radius (mm)	6.251 ± 0.156	6.470 ± 0.158	6.563 ± 0.140	6.650 ± 0.146	+0.399
BAD-D	6.854 ± 1.141	6.524 ± 1.101	6.370 ± 1.111	6.203 ± 1.139	−0.651

Note: The direction of the secondary parameters is concordant with reduced corneal deformability after treatment.

**Table 4:** Repeated-measures ANOVA and longitudinal Kmax-SP-A1 correlations.

Statistical Summary	F	Num DF	Den DF	*p*	Partial η^2^
UCVA	98.02	3	81	<0.001	0.784
BCVA	125.66	3	81	<0.001	0.823
Kmax	127.65	3	81	<0.001	0.825
SP-A1	537.51	3	81	<0.001	0.952
Thinnest pachymetry	71.43	3	81	<0.001	0.726
DA	1330.64	3	81	<0.001	0.980
A1T	1286.00	3	81	<0.001	0.979
A2T	305.10	3	81	<0.001	0.919
HC radius	879.77	3	81	<0.001	0.970
BAD-D	507.22	3	81	<0.001	0.949
Correlation timepoint	r			*p*	
Baseline	−0.773			<0.001	
6 months	−0.761			<0.001	
12 months	−0.714			<0.001	
24 months	−0.741			<0.001	

Note: Longitudinal findings were interpreted in relation to primary outcomes, clinical relevance, multiple testing, and eye-level clustering. Although all listed repeated-measures *p* values were <0.001, they should be considered descriptive inferential signals because the analysis did not model inter-eye dependence.

## Data Availability

The data supporting the findings of this study are available from the corresponding author on reasonable request, subject to institutional approval and patient confidentiality requirements.

## Abbreviations

BCVA, best-corrected visual acuity; CXL, corneal collagen cross-linking; KC, keratoconus; Kmax, maximum keratometry; logMAR, logarithm of the minimum angle of resolution; SP-A1, stiffness parameter at first applanation; UCVA, uncorrected visual acuity; UVA, ultraviolet A.
